# Bidirectional Mendelian Randomisation Analysis of Gastric Cancer and Depression: Evidence for the Causal Effect of Cancer on Depression

**DOI:** 10.62641/aep.v54i1.2097

**Published:** 2026-02-15

**Authors:** Juanjuan Liu, Yuehong Chen, Paiqi Zhang

**Affiliations:** ^1^Gastroenterology Department, Genertec Universal Crec Xi'an Hospital, 710054 Xi'an, Shaanxi, China

**Keywords:** gastric cancer, depression, genome-wide association studies, Mendelian randomisation

## Abstract

**Background:**

The relationship between gastric cancer and depression is an area of active investigation, and recent studies suggests a bidirectional association. Understanding this relationship is crucial for improving treatment approaches and mental well-being in patients with gastric cancer.

**Methods:**

We analysed the correlation between gastric cancer and depression, using data from Genome-Wide Association Studies. Causal links were explored using Mendelian randomisation (MR) and Gene Expression Omnibus.

**Results:**

Forward MR analysis identified 24 single nucleotide polymorphisms (SNPs) meeting the criteria for instrumental variables. The analysis provided evidence of a causal effect of gastric cancer on depression (odds ratio [OR]: 1.132, 95% confidence interval [CI]: 1.032–1.231). The reverse MR analysis, examining the potential causal effect in the opposite direction, identified 15 SNPs; however, no significant causal effect of depression on gastric cancer was detected (OR: 0.834, 95% CI: 0.504–1.380). Cross-pathway analysis identified 23 genes common to both conditions. Protein interaction network analysis of these shared genes revealed that lactoferrin, lipocalin-2 and matrix metalloproteinase-9 are potential key genes in the shared pathophysiology of both diseases.

**Conclusions:**

Our study demonstrates a causal effect of gastric cancer on depression, whereas depression does not exert a causal effect on gastric cancer. These findings provide evidence for targeted depression prevention strategies for patients with gastric cancer.

## Introduction

Gastric cancer is the fifth most frequently diagnosed cancer and the third leading cause of cancer deaths worldwide. The burden of gastric cancer is considerable, with an estimated 1.1 million new cases and 770,000 deaths in 2020 alone [1]. The high mortality rate associated with gastric cancer underscores the importance of research into its causes, prevention and treatment. Depression is a common mental disorder that affects millions of people worldwide. It is characterised by persistent sadness, loss of interest or pleasure, feelings of guilt or low self-worth, disturbed sleep or appetite, feelings of tiredness and poor concentration [2]. The relationship between depression and physical health conditions has been the subject of extensive research, and studies have revealed associations between depression and various diseases, including cardiovascular disease, diabetes and cancer [3, 4, 5].

Mechanistic studies have proposed several biological pathways through which depression might influence gastric cancer development. Chronic psychological stress and depression activate the sympathetic nervous system, elevating the levels of catecholamines (epinephrine and norepinephrine), which bind to β2-adrenergic receptors (ADRB2) on cancer cells [6]. Experimental evidence demonstrates that chronic stress promotes gastric cancer cell proliferation, invasion and metastasis through ADRB2 signalling pathways, and this effect is accompanied by the increased expression of vascular endothelial growth factor and matrix metalloproteinases (MMP-2, MMP-7 and MMP-9) [6]. Additionally, depression-associated immune dysregulation, characterised by chronic inflammation with elevated pro-inflammatory cytokines (Interleukin-6 (IL-6), Tumor Necrosis Factor-alpha (TNF-α) and C-reactive Protein (CRP)), may create a tumour-permissive microenvironment [7]. Depression is linked to unhealthy lifestyle behaviours, including smoking, excessive alcohol consumption and poor dietary patterns, which are established risk factors for gastric cancer [8, 9, 10]. These mechanisms have been primarily elucidated in experimental models, but their relevance to human gastric cancer aetiology remains unclear.

Previous studies have been exclusively observational, relying on epidemiological data from sampled populations. This approach is inherently limited: observational designs are susceptible to numerous confounding factors and cannot definitively establish causal relationships [11, 12]. Mendelian randomisation (MR) uses genetic variants as instruments to estimate the causal effect of an exposure on an outcome. By leveraging the random allocation of alleles at conception, MR helps reduce confounding and reverse causation common in observational studies [13, 14]. MR has been widely employed in studies on causal relationships among various diseases [15, 16, 17]. However, no study has systematically examined the bidirectional causal relationship between gastric cancer and depression with genetic approaches that can overcome the limitations of conventional observational studies.

Given conflicting evidence from observational studies and unclear causality, MR is crucial. Unlike conventional epidemiological approaches, MR leverages genetic variants randomly allocated at conception as instrumental variables, thereby minimizing confounding environmental factors and eliminating reverse causation [11, 18]. This approach is particularly valuable to investigations on depression–gastric cancer relationship, where bidirectional associations are plausible: depression may influence cancer risk through neuroendocrine, immune and behavioural pathways, and gastric cancer diagnosis and treatment-related distress may trigger depressive symptoms. Bidirectional MR analysis enables simultaneous assessment of both causal directions, providing comprehensive evidence of depression as a modifiable risk factor for gastric cancer prevention or primarily a consequence of cancer diagnosis [19]. Furthermore, MR can clarify whether the observed associations in prior studies reflect true causal effects or are artifacts of confounding and reverse causation. To the best of our knowledge, this study is the first to apply bidirectional two-sample MR to investigate the causal relationship between depression and gastric cancer.

Although epidemiological observations suggest a link between depression and gastric cancer, the causal relationship remains undetermined. This study integrates MR analysis of genome-wide association studies (GWAS) data with conventional epidemiological evidence to elucidate the mechanistic and epidemiological basis of their association and to provide theoretical support for simultaneous treatment strategies targeting both conditions.

## Materials and Methods

### Study Design

This study employed a two-sample MR framework to investigate the causal relationship between depression and gastric cancer. GWAS summary–level data for exposure and outcomes were obtained from FinnGen (Release 9). We assessed potential sample overlap between depression and gastric cancer GWAS cohorts from FinnGen. Given the distinct phenotype definitions, sample overlap was minimal and did not compromise the validity of our MR analysis. Gastric cancer and depression phenotypes were defined using standardised algorithms applied to Finnish national health registries, including the Hospital Discharge Register, Finnish Cancer Registry and Prescription Drug Purchase Register. Gastric Cancer: Cases were identified using ICD-10 code C16 (malignant neoplasm of stomach) from the FinnGen database. Depression: Cases were identified using ICD-10 codes F32 (depressive episode) and F33 (recurrent depressive disorder) from the FinnGen database. For gastric cancer, we utilised data from 1307 European (EUR) cases and 287,137 EUR controls. For depression, we included 43,280 EUR cases and 329,192 EUR controls. Potential causal associations between depression and gastric cancer were inferred through two-sample MR analysis using the aforementioned summary statistics.

### GWAS Data and Mendelian Randomisation Analysis

To explore the potential causal effect of depression on gastric cancer, we conducted two-sample MR analysis, using publicly available GWAS summary statistics. Single nucleotide polymorphisms (SNPs) significantly associated with depression (*p* < 5 × 10^-8^, F-statistic >10) were selected as instrumental variables. Linkage disequilibrium was addressed using the clumping function (r^2^ < 0.0001) in PLINK software (v1.90, Center for Human Genetic Research, Massachusetts General Hospital, Boston, MA, USA; Broad Institute of MIT and Harvard, Cambridge, MA, USA), and SNPs absent in the gastric cancer GWAS dataset were excluded. The final set of SNPs was reconciled between exposure and outcome datasets.

MR analysis was performed under three core assumptions required for valid causal inference: (1) relevance assumption—the selected genetic variants are robustly associated with exposure (depression); (2) independence assumption—genetic variants are independent of confounding variables that affect exposure and outcome; and (3) exclusion restriction assumption—genetic variants influence the outcome (gastric cancer) only through the exposure pathway, and horizontal pleiotropy is absent. The inverse-variance weighted (IVW) method was used as the primary approach to estimate causal effects. The IVW method, which weighs each SNP–outcome association by the inverse of the variance, was used as the primary approach to estimate causal effects. This method assumes balanced horizontal pleiotropy and provides a weighted linear regression estimate under the fixed-effects model. Results are presented as odds ratios (OR) with 95% confidence intervals (CIs). Additional MR methods, including MR–Egger, weighted median and simple mode, were employed to ensure robustness of the findings and to address potential violations of instrumental variable assumptions. The complete bidirectional MR analytical framework is summarised in **Supplementary Fig. 1**.

### Statistical Analysis

The validity and stability of the MR results were assessed using several sensitivity analyses. Heterogeneity among SNPs was evaluated using Cochran’s Q statistic, and potential horizontal pleiotropy was tested using the MR–Egger intercept. Whether any single SNP disproportionately influenced the causal estimates was determined through leave-one-out analysis. To assess potential reverse causality, we performed bidirectional MR analysis and evaluated gastric cancer as an outcome and an exposure. Adjustments for multiple testing were performed through Bonferroni correction, and the directionality of the causal pathway was confirmed through Steiger filtering. All analyses were conducted using TwoSampleMR package (v0.5.6, MRC Integrative Epidemiology Unit, University of Bristol; Bristol, UK) in R (v4.1.0 R Foundation for Statistical Computing; Vienna, Austria).

### Gene Expression Analysis

Gene expression data for gastric cancer and depression were obtained from the Gene Expression Omnibus database. Two datasets with large sample sizes were selected: GSE66229 and GSE98793. GSE66229 consists of 100 normal and 200 tumour gastric cancer tissue gene expression data, whereas GSE98793 includes 64 normal and 64 case whole blood gene expression data for depression. These datasets are suitable for cross-analysis for identifying co-published genes. Differential expression analysis was conducted using limma package (v3.54.2, Walter and Eliza Hall Institute of Medical Research; Melbourne, Victoria, Australia.), with a threshold of |logFC| >0.3 and *p* < 0.05. We utilised the STRING database (v12.0, University of Zurich and SIB Swiss Institute of Bioinformatics, Zurich and Lausanne, Switzerland) to construct a protein–protein interaction network for genes with common differential expression and screen for key proteins. Furthermore, functional enrichment analysis was performed on the differentially expressed genes with ClusterProfiler (v4.6.2, Southern Medical University, Guangzhou, China). GSE134520 is a single-cell sequencing dataset containing 13 gastric cancer tissues. After performing routine cell annotation on the single-cell sequencing data, we visualise the expression characteristics of the top five genes in different cells with UMAP plots.

## Results

### Genome Associations Between Gastric Cancer and Depression

**Fig. 1.**
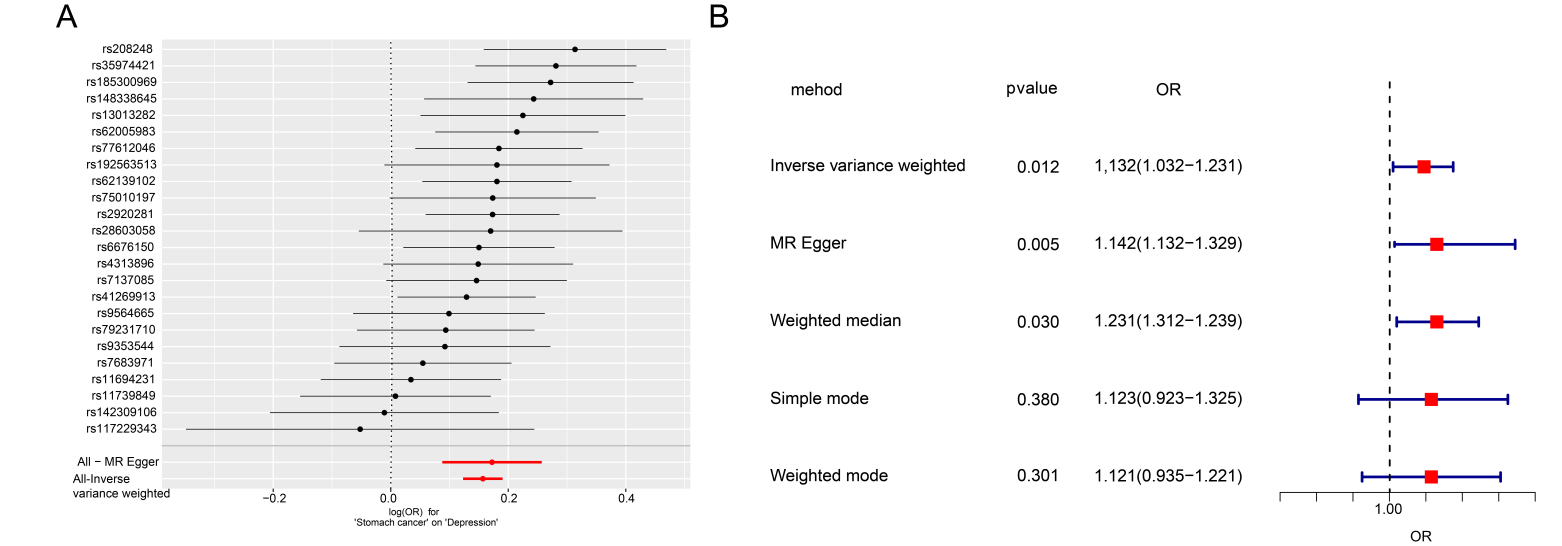
**Analysis of the association between gastric cancer as an exposure factor and depression**. (A) All mutation sites related to gastric cancer. (B) OR value of Mendelian model corrected based on multiple algorithms. OR, Odds Ratio; MR, Mendelian randomisation.

Utilizing data from GWAS for gastric cancer and depression, we conducted a two-sample MR analysis to investigate the potential causal relationship between the two conditions. The analysis identified 24 SNPs that are significantly associated with gastric cancer and meet the criteria for instrumental variables (Fig. 1A). Using the IVW method as the primary analysis approach, we observed that gastric cancer was significantly associated with increased risk of depression (OR: 1.132, 95% CI: 1.032–1.231; Fig. 1B). However, sensitivity analyses employing alternative MR methods (MR–Egger, weighted median and weighted mode) demonstrated consistent estimates with overlapping CIs, although with slightly attenuated effect sizes, suggesting the robustness of the primary findings. Among the 24 SNPs identified in the forward MR analysis, 21 SNPs were associated with annotated genes (Table 1). The remaining three SNPs located in intergenic regions were retained in the MR analysis as valid instrumental variables but were not included in the annotated SNP table. Detailed SNP information, including all instrumental variables used in the forward MR analysis, is presented in Table 1.

**Table 1.** **Basic information about key SNPs**.

Genes	rsid	Chromosome	Position
*LINC01250*	rs11694231	2	3105099
*BAIAP2L2*	rs117229343	22	38068083
*KCTD16*	rs11739849	5	144206059
*CAPN13*	rs13013282	2	30661317
*FRMD6*	rs142309106	14	51442103
*C10orf113*	rs148338645	10	21034651
*MIR1252*	rs185300969	12	79426876
*C2orf61*	rs192563513	2	47094579
*PTPRT*	rs208248	20	42788818
*NKX6-1*	rs28603058	4	84417777
*ARC*	rs2920281	8	142679026
*SCAF4*	rs35974421	21	31727155
*ADAR*	rs41269913	1	154489004
*CCBE1*	rs4313896	18	59461626
*WDR72*	rs62005983	15	53768729
*ALK*	rs62139102	2	29993760
*ADAM15*	rs6676150	1	155151361
*PTHLH*	rs7137085	12	27932196
*EVA1A*	rs77612046	2	75448369
*CNOT4*	rs79231710	7	135311860
*ATXN8OS*	rs9564665	13	70206148

**Fig. 2.**
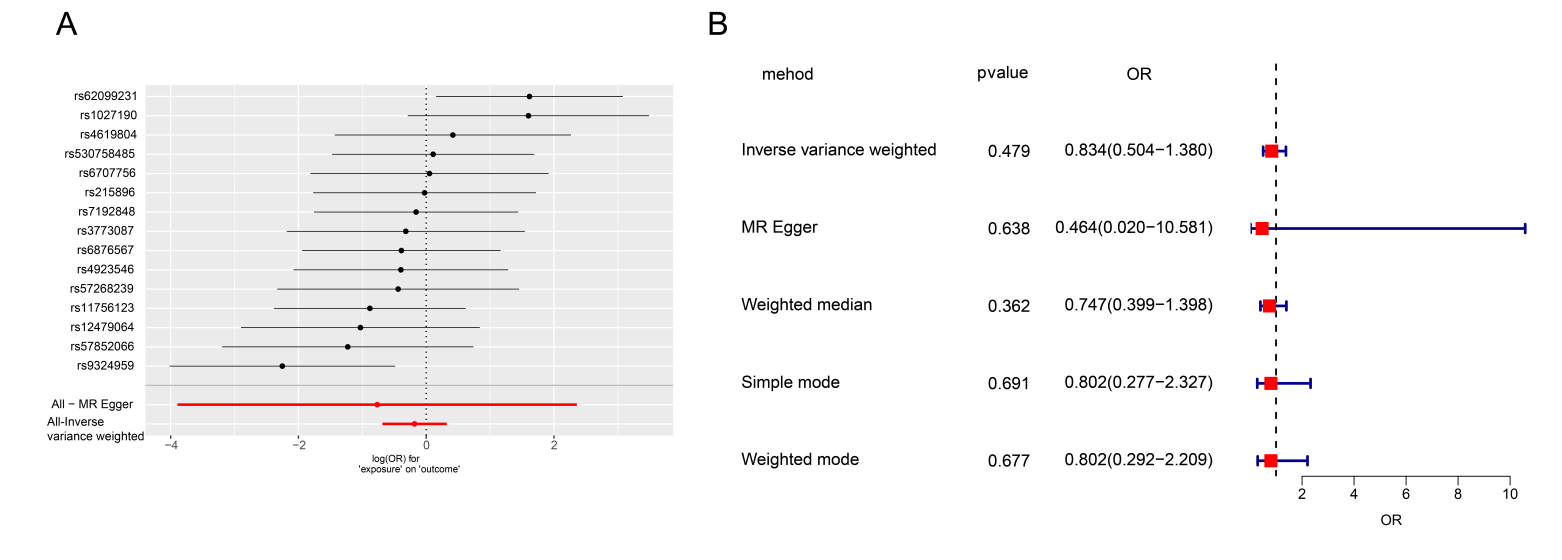
**Analysis of the association between depression as an exposure factor and gastric cancer**. (A) All mutation sites related to depression. (B) OR value of Mendelian model corrected based on multiple algorithms. OR, Odds Ratio; MR, Mendelian randomisation.

The potential causal effect of depression on gastric cancer was investigated through reverse MR analysis. A total of 15 SNPs associated with depression were identified as instrumental variables (Fig. 2A). When the IVW method was applied, depression was not found to have a statistically significant causal effect on gastric cancer risk (OR: 0.834, 95% CI: 0.504–1.380; Fig. 2B). Sensitivity analyses using complementary MR methods (MR–Egger, weighted median and weighted mode) yielded consistent null findings, and CIs consistently encompassed the null value, further supporting the absence of a significant causal association in this direction. These results indicate that the observed association between depression and gastric cancer is primarily driven by the effect of gastric cancer on depression development, rather than the opposite. To determine the stability of our results, we evaluated model robustness by heterogeneity test. The results were relatively stable, regardless of whether they were positive or reverse (Table 2).

**Table 2.** **MR heterogeneity test**.

Outcome	Exposure	Method	Q	Q_df	Q_pval
Gastric cancer	Depression	MR Egger	17.898818	13	0.161399213
Gastric cancer	Depression	Inverse variance weighted	18.08970967	14	0.202725492
Depression	Gastric cancer	MR Egger	26.08814346	22	0.247916833
Depression	Gastric cancer	Inverse variance weighted	26.27335866	23	0.288131907

MR, Mendelian randomisation.

### Gene Expression Associations Between Gastric Cancer and Depression

**Fig. 3.**
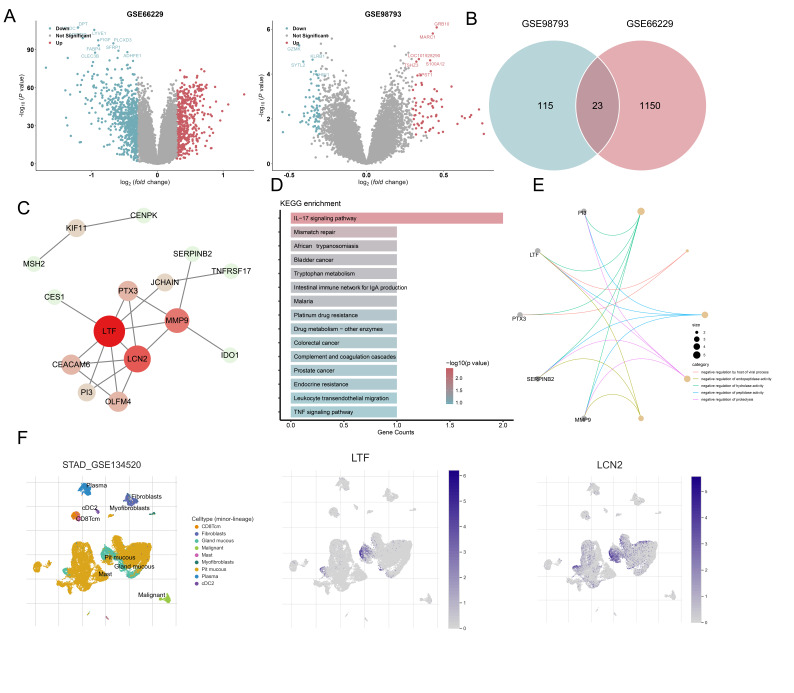
**LTF, LCN2 and MMP9 are key differential genes in gastric cancer and depression**. (A) Volcano plot of differential expression analysis of gastric cancer and depression datasets. (B) Intersection VENN plot of two disease differential genes. (C) Differential gene–protein interaction network diagram. (D) Cross-gene KEGG analysis histogram. (E) GO analysis network diagram of intersection genes. (F) Expression of key genes in gastric cancer single cell data. KEGG, Kyoto Encyclopedia of Genes and Genomes; GO, Gene Ontology; LTF, lactoferrin; LCN2, lipocalin-2; MMP9, matrix metalloproteinase-9.

To further determine the pathogenesis similarity between the two, we performed differential expression analysis, using two sequencing data sets for gastric cancer and depression. A total of 406 and 840 differentially expressed genes were identified through differential expression analysis using GSE66229. Further analysis of GSE98793 data revealed that 96 genes were highly expressed in patients with depression and 107 genes were highly expressed in normal controls (Fig. 3A). Cross-analysis of these differentially expressed genes showed that 23 genes were shared between the two conditions (Fig. 3B). To better understand the interaction between these genes, protein interaction analysis was conducted, revealing that lactoferrin (LTF), lipocalin-2 (LCN2) and matrix metalloproteinase-9 (MMP9) play key roles in the overall interaction network (Fig. 3C). Functional enrichment analysis of these intersecting genes showed a significant association with the IL17 signalling pathway (Fig. 3D). Additionally, Gene Ontology analysis of these genes revealed a strong correlation with negative regulation of endopeptidase activity (Fig. 3E). Finally, examination of single-cell sequencing data for gastric cancer showed that LTF and LCN2 were predominantly expressed in the mucous gland (Fig. 3F).

## Discussion

Gastric cancer, a leading cause of cancer-related mortality worldwide, is a multifactorial disease influenced by a complex interplay of genetic, environmental and lifestyle factors. The role of psychological factors, particularly depression, has emerged as a potential contributor to the risk and progression of gastric cancer [20, 21, 22]. This study explores the association between depression and gastric cancer, using GWAS and RNA-seq data to elucidate the causal relationship between these conditions.

Through MR analysis, our research establishes a unidirectional causal pathway in the gastric cancer–depression relationship: gastric cancer occurrence significantly increases depression risk, whereas pre-existing depression does not causally elevate gastric cancer risk. This finding aligns with recent large-scale MR studies demonstrating no causal link between psychiatric disorders and gastric cancer incidence and longitudinal cohort evidence showing that depressive symptoms fail to predict subsequent cancer development across 778,802 person-years of observation. The forward causality from gastric cancer to depression is mechanistically supported by multiple converging pathways. First, gastric cancer diagnosis and treatment impose severe psychological burden, and up to 57% of patients, particularly those with advanced disease, experienced clinically relevant depressive symptoms [23]. This distress stems from disease-specific stressors, including fear of mortality, aggressive treatment side effects (weight loss, digestive dysfunction and stoma formation), financial strain and body image disturbance unique to gastrointestinal malignancies. Second, biological mechanisms establish gastric cancer as a driver of neuropsychiatric dysfunction: tumour-associated chronic stress activates the sympathoadrenal axis, increasing circulating catecholamines that bind to ADRB2 [24]. ADRB2 activation triggers the release of pro-inflammatory cytokines (IL-6 and TNF-α), creating a systemic neuroinflammatory environment [25]. Elevated intratumour norepinephrine concentrations have been documented in cancer patients with high biobehavioural risk profiles, directly linking tumour biology to neurotransmitter dysregulation and depressive symptomatology [26]. Third, this inflammatory cascade disrupts central neurotransmitter balance and induces behavioural depression symptoms while simultaneously promoting a pro-tumourigenic microenvironment, forming a neuroimmune–oncogenic feedback loop [27]. Conversely, the absence of reverse causality is robustly supported: bidirectional MR analyses across 17 cancer types detected no evidence that genetically predicted depression increases cancer risk after controlling for horizontal pleiotropy and confounders. Multiple sensitivity analyses using weighted median and MR–Egger methods confirmed psychiatric disorders, including major depressive disorder, show no causal relationship with digestive tract cancer development. Large-scale prospective cohort data further validate that depressive symptoms at baseline fail to predict incident gastric or other site-specific cancers when properly adjusting for reverse causation bias. These converging lines of evidence, that is, MR eliminates confounding, longitudinal cohorts controlling temporal sequence, and mechanistic studies reveal tumour-driven pathophysiology and collectively establish that gastric cancer functions as the primary aetiological driver of depression in this relationship, rather than depression serving as a risk factor for gastric cancer development.

Two SNPs of interest in the context of gastric cancer and depression are RS11739849 and RS11694231. RS11739849 is an SNP located in an intronic region upstream of the *KCTD16* gene. The *KCTD16* gene, also known as potassium channel tetramerisation domain 16, is involved in protein homologous oligomerisation and has a potential role in regulating the upstream or internal signalling pathways of G protein-coupled receptors. It is located in cell projections and may be a component of receptor complexes [28, 29, 30]. By contrast, RS11694231 is a SNP located in an intronic region of a long non-coding RNA called LINC01250. The relationship between LINC01250 and gastric cancer has been extensively studied. In a review on genetic polymorphisms associated with gastric cancer risk, LINC01250 was identified as one of the genes linked to this disease. Additionally, studies have suggested a potential genetic overlap between LINC01250 and depression, specifically in relation to Alzheimer’s disease [31]. However, further research is needed to fully understand the mechanisms underlying these two SNPs and their potential role in the association between depression and gastric cancer.

To further explore the association between the two diseases, we used RNA-seq data from both diseases for cross-analysis to identify differentially expressed genes related to both diseases. After analysis, we determined a total of three key genes. Lactoferrin (LTF), an iron-binding glycoprotein with multifunctional roles in innate immunity and tumour suppression, exhibits significantly downregulated expression in gastric cancer tissues, with approximately 20-fold reduction compared with adjacent non-cancerous tissues. Mechanistically, LTF suppresses gastric cancer progression by modulating the MAPK signalling pathway, particularly through p38, JNK and c-Jun downregulation. Accumulating evidence indicates that LTF deficiency during early development increases susceptibility to depressive phenotypes in adulthood through dysregulation of the microbiota–gut–brain axis and neuroinflammatory pathways, whereas LTF supplementation alleviates depressive symptoms by inhibiting TLR4-NF-κB signalling and promoting neuronal proliferation through ERK1/2 phosphorylation. LCN2, a secreted glycoprotein involved in iron homeostasis and innate immune responses, demonstrates tumour-suppressive functions in gastric cancer through the autocrine inhibition of the 24p3R/JNK/c-Jun/SPARC axis, and elevated LCN2 expression correlates with reduced tumour grade and improved prognosis [32, 33]. In psychiatric disorders, elevated serum LCN2 levels have been associated with anxiety and depression, and LCN2 knockout mice exhibit anxiety- and depression-related behaviours alongside hippocampal neuronal morphology alterations and synaptic impairment. MMP9, a key extracellular matrix-degrading enzyme, emerges as a potential molecular link between gastric cancer and depression through shared inflammatory pathways. MMP9 is significantly elevated in gastric cancer tissues where it promotes tumour progression and metastasis and elevates serum MMP9 levels contribute to depression pathophysiology through blood–brain barrier disruption and neuroinflammation. The bidirectional association between these conditions may be mediated by MMP9-driven inflammatory mechanisms, wherein cancer-induced MMP9 elevation promotes systemic inflammation and depression development. Depression-associated MMP9 upregulation enhances tumour microenvironment remodelling. This shared mechanistic pathway implicates MMP9 as a therapeutic target and potential biomarker for gastric cancer–related depression [34, 35, 36]. The integrated evidence suggests that LTF, LCN2 and MMP9 constitute potential molecular links between inflammatory processes, cancer progression and neuropsychiatric manifestations, warranting further investigation of their mechanistic roles in comorbid conditions.

This study has important limitations to consider. First, findings are limited to individuals of European ancestry and may not generalise to other ethnic groups. Second, although MR sensitivity analyses were performed, strict adherence to all MR assumptions cannot be fully guaranteed. Third, a notable discrepancy exists between the 24 SNPs identified in GWAS and the 23 differentially expressed genes identified in transcriptomic analysis, suggesting that disease-associated genetic variants operate through regulatory mechanisms not directly captured by bulk gene expression analysis. Many SNPs may function as expression quantitative trait loci affecting gene regulation through distant regulatory regions or cell-type-specific mechanisms, and the identified differentially expressed genes reflect altered steady-state expression influenced by multiple post-transcriptional regulatory layers. This disjunction highlights unexplored regulatory complexity requiring future multi-omics studies integrating eQTL mapping, enhancer annotation and colocalisation analysis. Fourth, many disease-associated SNPs did not directly correlate with the expression of the key hub genes (LTF, LCN2 and MMP9), indicating that they likely exert effects through indirect regulatory mechanisms, including non-coding RNA regulation or chromatin-level effects. This result necessitates future studies employing fine-mapping, tissue-specific eQTL analysis, and three-dimensional chromatin mapping. Fifth, analyses were not stratified by specific gastric cancer subtypes or anatomical location, and histological heterogeneity may influence the gastric cancer–depression relationship. Finally, cross-disease transcriptomic comparisons may be confounded by batch effects from heterogeneous microarray platforms and normalisation procedures, warranting validation through prospective cohorts with standardised sequencing methods.

## Conclusions

This study establishes gastric cancer as a causal driver of depression through integrated MR and transcriptomic analysis. Bidirectional MR analysis demonstrates that gastric cancer considerably increases depression risk, and pre-existing depression does not causally affect gastric cancer development, indicating a unidirectional causal pathway. Gene expression profiling identified 23 differentially expressed genes common to both conditions. LTF, LCN2 and MMP9 emerging as hub genes in protein–protein interaction networks. These genes converge on IL-17 signalling and inflammatory pathways, and MMP9 simultaneously promotes tumour invasion and blood–brain barrier disruption-induced neuroinflammation. Additionally, LTF and LCN2 expression in mucous gland cells, supporting tissue-specific mechanisms linking gastric cancer to depression. These findings provide an evidence-based foundation for implementing systematic depression screening in patients with gastric cancer and identify LTF, LCN2 and MMP9 as potential biomarkers and therapeutic targets for integrated oncological and psychiatric intervention.

## Preprint

A preprint version of this manuscript was previously published on Research Square (https://doi.org/10.21203/rs.3.rs-4194989/v1) on April 29, 2024.

## Availability of Data and Materials

All data come from the publicly available datasets in present study. The datasets used and/or analyzed during the current study are available from the corresponding author on reasonable request.

## Supplementary Material

Supplementary material associated with this article can be found, in the online version, at https://doi.org/10.62641/aep.v54i1.2097.
