# Supplementary material for: Bidirectional Mendelian Randomisation Analysis of Gastric Cancer and Depression: Evidence for the Causal Effect of Cancer on Depression
Source: Actas Esp Psiquiatr. 2026 Feb 15;54(1):97–106. doi: 10.62641/aep.v54i1.2097 (PMC12946735; doi:10.62641/aep.v54i1.2097)
Supplement: Supplementary file 1 [file ActEsp-54-1-97-106-s1.zip › Supplementary Fig.1.docx]

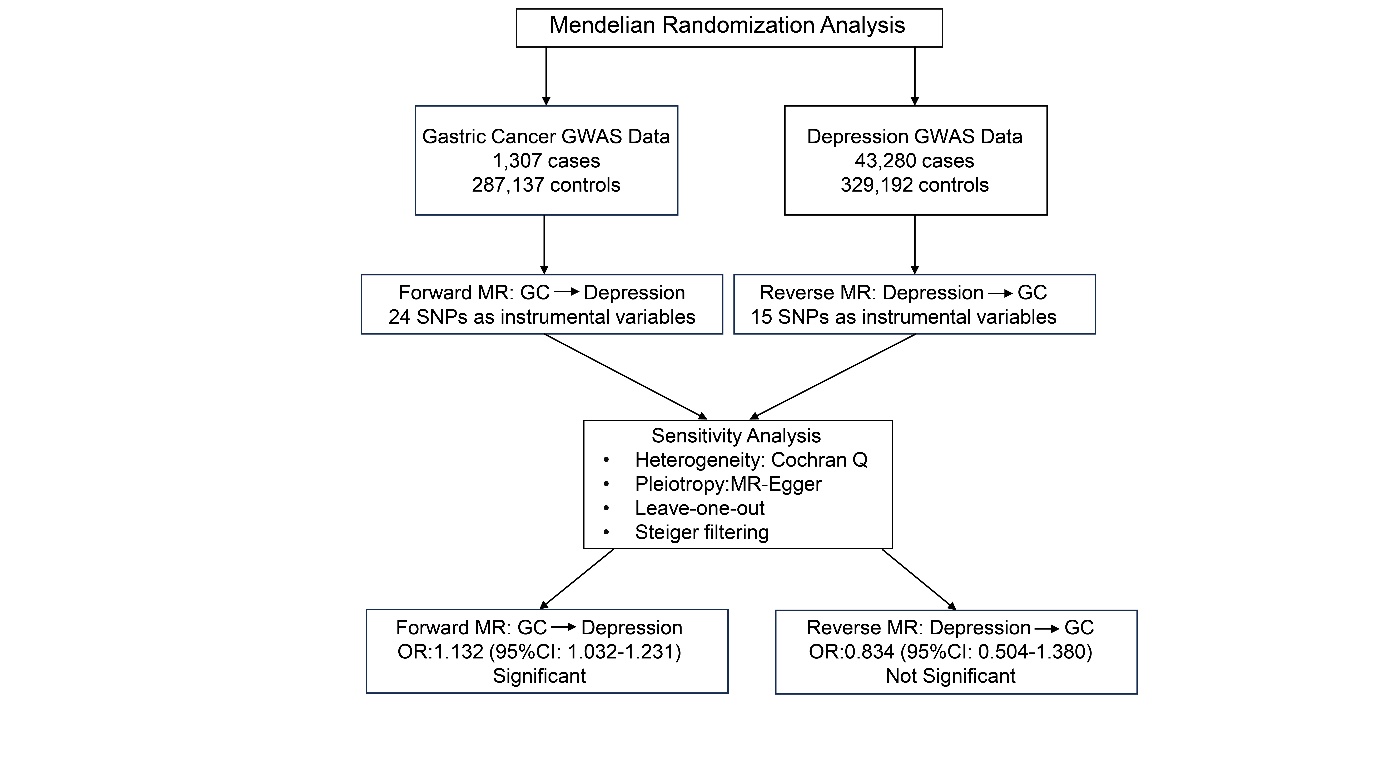


**Supplementary Fig. 1. Flowchart of the bidirectional Mendelian randomisation analysis.** GWAS, genome-wide association studies; MR, Mendelian randomization; OR, odds ratio; CI, confidence interval; GC, Gastric Cancer.
